# Supplementary material for: Charge carrier mobility in thin films of organic semiconductors by the gated van der Pauw method
Source: Nat Commun. 2017 Apr 11;8:14975. doi: 10.1038/ncomms14975 (PMC5394272; doi:10.1038/ncomms14975)
Supplement: Supplementary Information — Supplementary Figures, Supplementary Table 1 and Supplementary Note 1 [file ncomms14975-s1.pdf]

Supplementary Information

Supplementary Figures

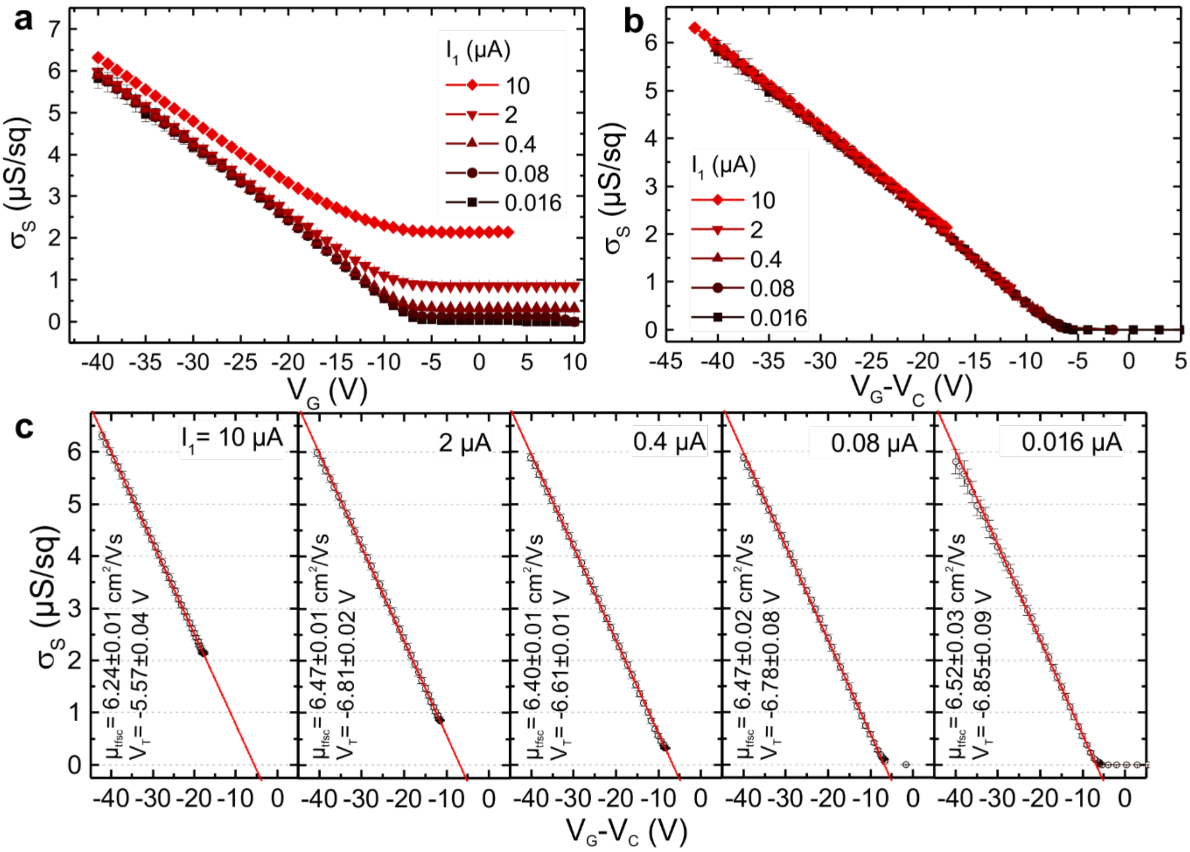

Figure 1: Sheet conductance σ_s of the C10-DNTT gVDP device. The gVDP characteristic of this device is presented in Fig. 2c of the main text. Here σ_s is plotted as a function of (**a**) V_G and (**b**) V_G-V_C for direct comparison. In (a), current lines are separated and show non-linearity. In (b), the same data falls along the same straight line that can easily be fitted by Equation 3 from the main text in order to extract meaningful electrical data. This validates the use of Eq. 3 as an adequate model for gVDP operation. (**c**) Same as (b) but detailed current by current. The error bars on this data are calculated from the spread in the eight measurements performed around the gVDP structure. Red lines are linear fits. Mobility μ_{tfsc} is given by the slope of the fit divided by the gate dielectric capacitance (= 2.78 x 10^{-8} Fcm^{-2} for 124 nm SiO_2), following Eq. 3 in the main text. V_T is obtained from the intercept of the fit with the X-axis.

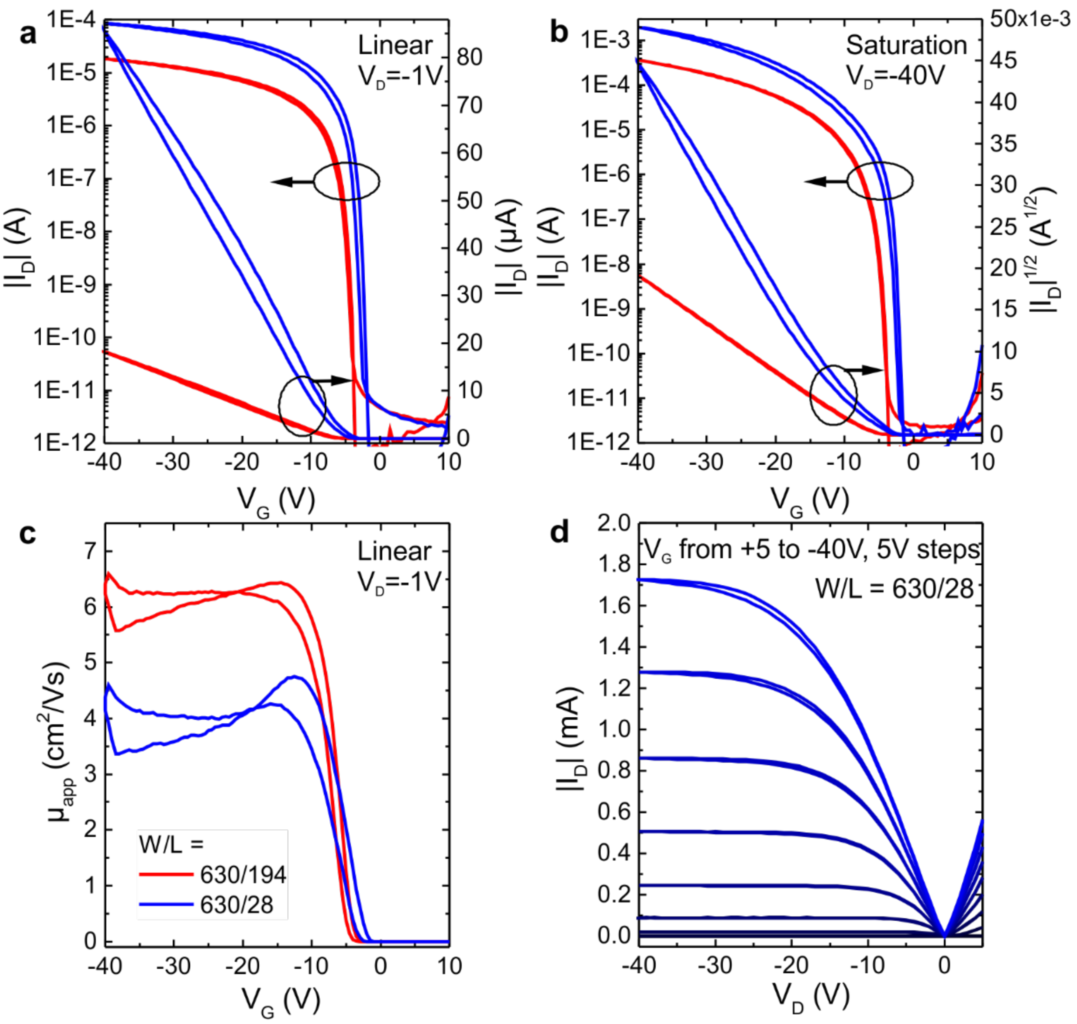

Figure 2: Characteristics of some TFTs from the C_{10}-DNTT TLM structure studied in Fig. 3 of the main text. Red and blue curves are characteristics of the TFT with channel length L = 194 µm and 28 µm, respectively. Transfer curves measured in (**a**) linear and (**b**) saturation regime. (**c**) Apparent mobility μ_{app} extracted from the linear regime transfer curves using the conventional transconductance method. (**d**) Output curves of the short channel device.

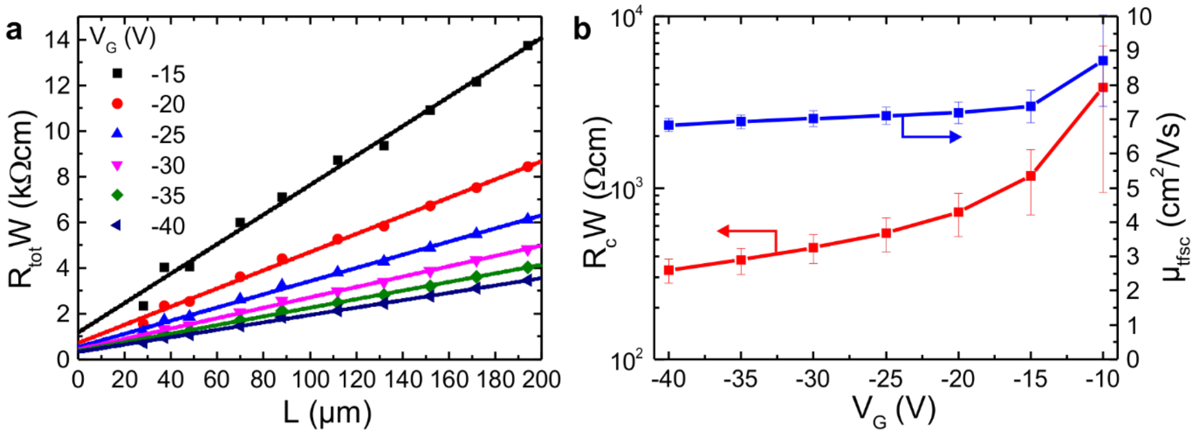

Figure 3: TLM analysis of the C_{10}-DNTT TLM structure. (**a**) Width-corrected total device resistance $R_{tot}W$ as a function of TFT channel length L, for different gate voltages V_G. The lines are linear fits used to extract R_c and μ_{tfsc}. (**b**) R_c and μ_{tfsc} from the TLM analysis as a function of V_G.

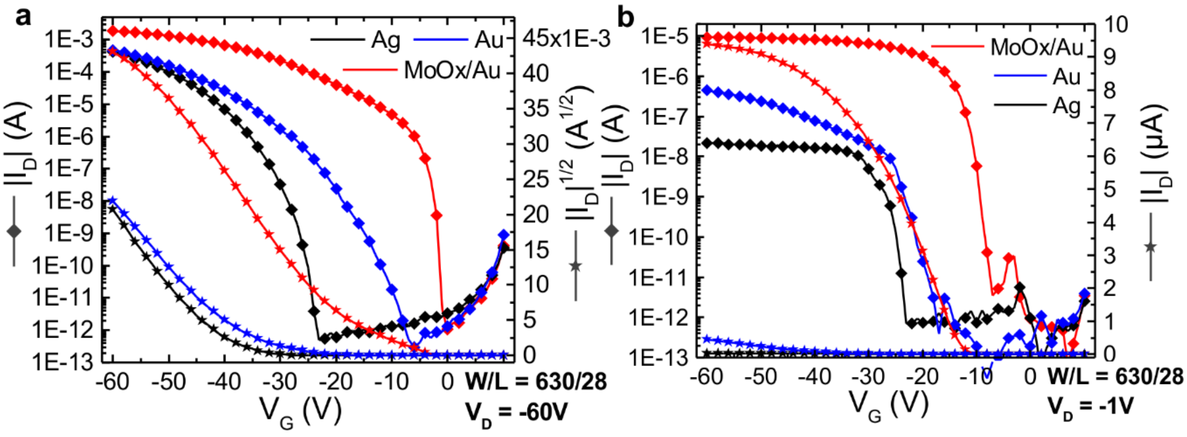

Figure 4: Transfer characteristics of TFTs strongly affected by contact resistance. The TFTs (W/L = 630/28 µm) are based on thin evaporated films of C_8-BTBT and three different top contact materials. (**a**) Saturation regime. (**b**) Linear regime.

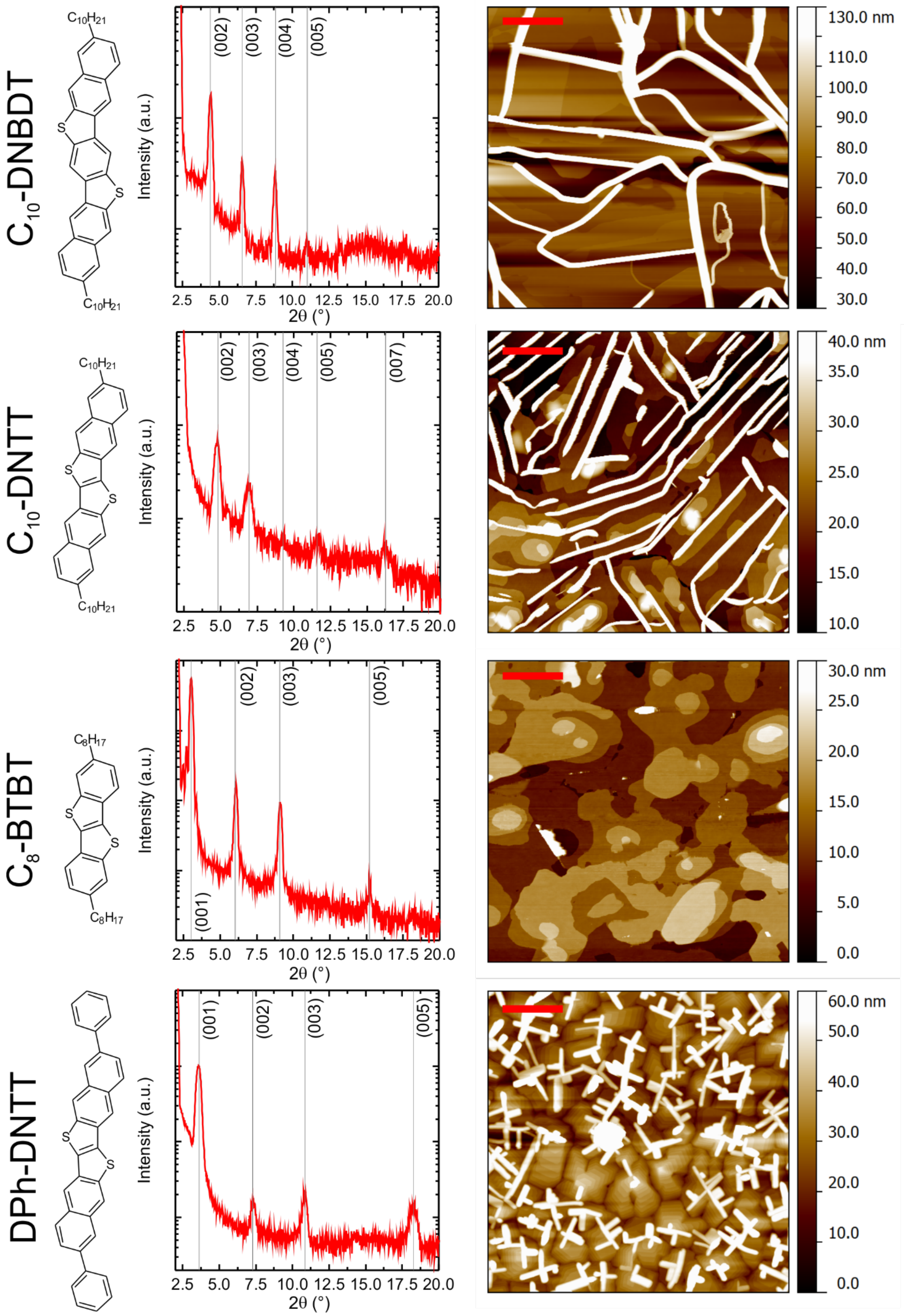
C10-DNBDT
C10H21
C10H21
Intensity (a.u.)
(002)
(003)
(004)
(005)
2.5 5.0 7.5 10.0 12.5 15.0 17.5 20.0
2θ (°)
130.0 nm
110.0
100.0
90.0
80.0
70.0
60.0
50.0
40.0
30.0
C10-DNTT
C10H21
C10H21
Intensity (a.u.)
(002)
(003)
(004)
(005)
(007)
2.5 5.0 7.5 10.0 12.5 15.0 17.5 20.0
2θ (°)
40.0 nm
35.0
30.0
25.0
20.0
15.0
10.0
C8-BTBT
C8H17
C8H17
Intensity (a.u.)
(001)
(002)
(003)
(005)
2.5 5.0 7.5 10.0 12.5 15.0 17.5 20.0
2θ (°)
30.0 nm
25.0
20.0
15.0
10.0
5.0
0.0
DPh-DNTT
Intensity (a.u.)
(001)
(002)
(003)
(005)
2.5 5.0 7.5 10.0 12.5 15.0 17.5 20.0
2θ (°)
60.0 nm
50.0
40.0
30.0
20.0
10.0
0.0

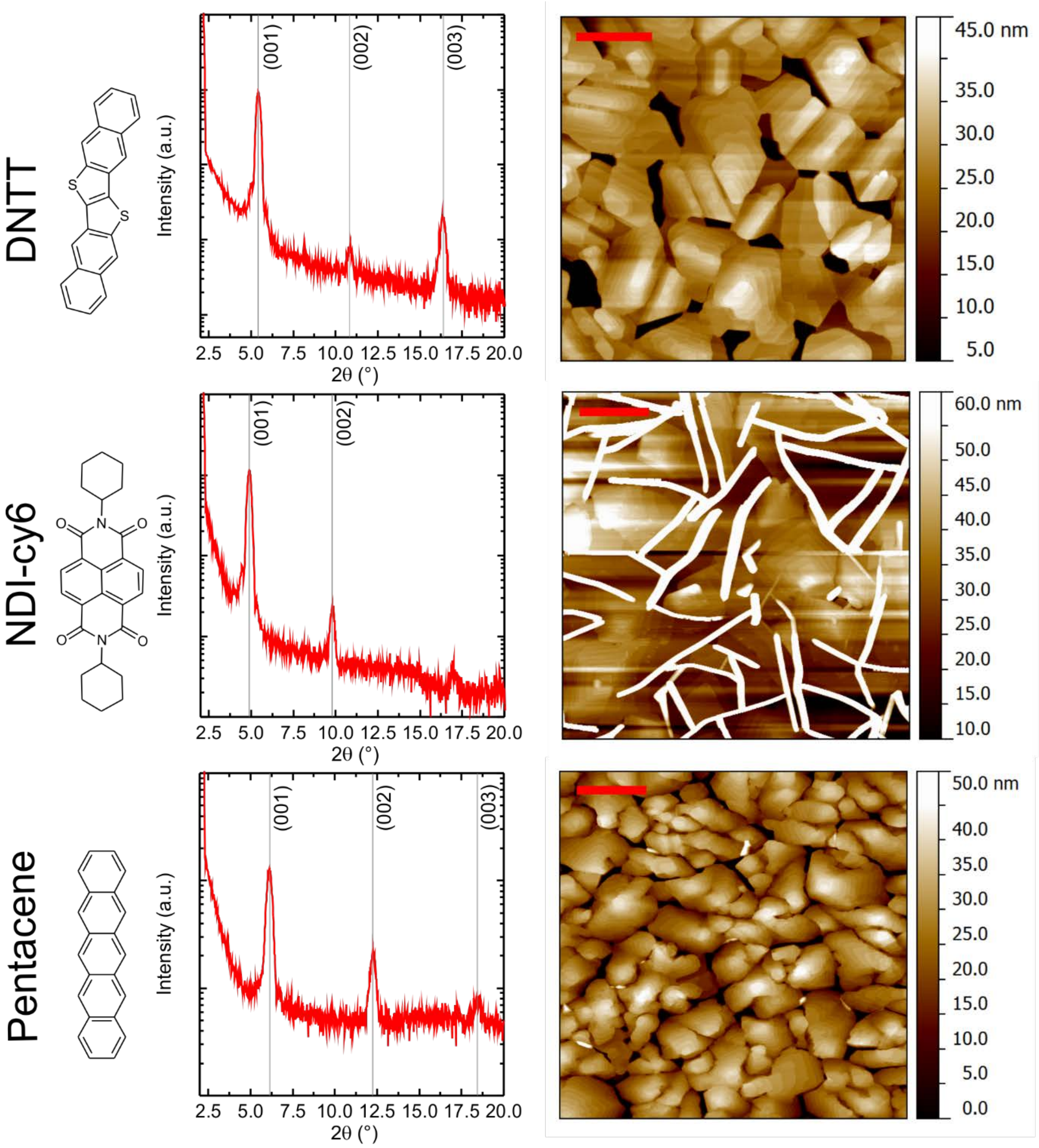

Figure 5: Out-of-plane XRD spectra and AFM images of the optimized thin films of the seven organic semiconductors examined in this study. All scale bars are 1 μm long. All films were vacuum evaporated with a thickness of ~30 nm using the optimized growth conditions of Table 3 in the main text. Data extracted from the spectra and images is presented in Supplementary Table 1 and discussed in the main text and in Supplementary Note 1.

Supplementary Table

Table 1. Thin film microstructural characteristics extracted from XRD spectra and AFM images.

Organic semiconductor	*d-spacing XRD (Å)*	*Terrace height AFM (Å)*	*Grain diameter AFM (µm)*
C_{10}-DNBDT	40.2 ± 0.1	40 ± 6	> 5
C_{10}-DNTT	37.6 ± 0.4	38 ± 2	> 1
C_8-BTBT	29.1 ± 0.1	29 ± 3	> 2
DPh-DNTT	24.4 ± 0.2	26 ± 3	~0.5
DNTT	16.3 ± 0.1	16 ± 2	~1
NDI-cy6	18.0 ± 0.1	17 ± 3	>1
Pentacene	14.4 ± 0.1	15 ± 2	~0.5

Supplementary Note 1

Morphological and microstructural characterization. For the needs of morphological and microstructural characterization, thin (~30 nm) films of the seven semiconductors examined in this study were deposited on pretreated Si/SiO_2 substrates with the growth conditions given in Table 3 of the main text. Out-of-plane X-ray diffraction (XRD) characterization was performed on a PANalytical X'Pert Pro Materials Research Diffractometer using Cu Kα radiation. Atomic Force Microscopy (AFM) images were acquired on a Bruker Dimension Edge scanning probe in tapping mode.

The peaks in out-of-plane XRD spectra are characteristic of crystallographic planes lying parallel to the substrate. The distance between each plane, i.e. the d-spacing, is obtained using Bragg's law and reported in Supplementary Table 1. For all semiconductors, the d-spacing roughly corresponds to the molecular long axis, confirming that molecules adopt a two dimensional layer-by-layer microstructure with molecules mostly standing up on their long axis. This growth habit is confirmed by the AFM images that reveal terraced patterns for all

semiconductors. The height of the terrace steps estimated from the AFM images is reported in Supplementary Table 1 and corresponds to the d-spacings from XRD. The two-dimensional grain diameters given in Supplementary Table 1 are roughly estimated from the AFM images. Note that these apparent grains do not necessarily correspond to the grains present in the first monolayer formed on the substrate in early stages of growth.

In most cases, tall three-dimensional structures, mostly needles, appear on the AFM image and may also results in additional XRD peaks at higher 2θ angles. These structures results from a roughening of the film beyond a certain thickness threshold. The crystallographic orientation of these needles differs from the orientation of the underlying two-dimensional film. In these needles, molecules are no longer necessarily standing on their long axis. It remains debated how much these three-dimensional structures participate in the lateral transport of charge carriers.
